# Home Advantage Perceptions in Elite Handball: A Comparison Among Fans, Athletes, Coaches, and Officials

**DOI:** 10.3389/fpsyg.2021.782129

**Published:** 2022-01-24

**Authors:** Lael Gershgoren, Orr Levental, Itay Basevitch

**Affiliations:** ^1^School of Behavioral Sciences, The College of Management Academic Studies, Rishon LeTsiyon, Israel; ^2^Department of Physical Education, Tel Hai College, Upper Galilee, Israel; ^3^School of Social and Behavioral Sciences, Northcentral University, California, CA, United States

**Keywords:** home advantage, sport, crowd effect, fans, spectators

## Abstract

Home advantage in sports has been extensively researched in the academic literature over the past five decades. A review of the literature reveals several factors that consistently underly this phenomenon. One of the most documented is the home crowd effect. While the crowd effect on the results has been widely researched considering noise, size, and density, there are conflicting findings of the effect and its extent. Furthermore, the perceptions of fans, athletes, coaches, and officials of the causes of home advantage in general and the crowd effect in particular, remain marginal. This is especially important in the face of significant regulation changes in the stands caused by the COVID-19 pandemic. The current study, therefore, examined the perceptions of fans, athletes, coaches, and officials of the Israeli handball premier league regarding fans’ contribution to the home advantage phenomenon along with other factors (e.g., travel and officiating). A questionnaire examining perceptions regarding home advantage was distributed to 232 Israeli participants (117 fans, 59 players, 26 coaches, and 30 officials). Results, based on MANOVA, ANOVA, and post-hoc analyses, indicated significant differences in participants’ perceptions of the different factors in general and the crowd factor in particular. Overall, the crowd was perceived as the most important factor contributing to the home advantage phenomenon (*M* = 5.7). Furthermore, fans perceived their contribution (i.e., the crowd) significantly higher than the rest of the participants (*p* = 0.001; i.e., players, coaches, and officials). On the other hand, officials ranked their contribution to the home advantage effect as low as well as significantly under ranked their contribution in comparison to the other groups (*p* < 0.001). This result suggests that officials perceive themselves as relatively robust to the crowd effect compared to the other participants. Additional results are discussed in light of existing gaps in the literature on the home advantage phenomenon. Alongside the theoretical contribution, these findings contribute to applied implications of increasing the home advantage effect when playing at home and negating the home advantage when playing away.

## Introduction

“Home advantage” in sports is a phenomenon well documented and well researched. The term “Home advantage” is defined as “the consistent finding in which the home teams in sport competitions win over 50% of the games played under a balanced home and away schedule” (Courneya and Carron, 1992, p. 13). Jamieson (2010), in her meta-analysis, denoted that approximately 60% of competitive sport games are won by the home team, regardless of sport’s type (i.e., individual or team) or level of competition (i.e., amateur, professional, or elite). This finding is consistent with the current body of research and suggests that the home advantage ratio is fixed around 60% (Strauss and Bierschwale, 2008; Gómez et al., 2011; Gutierrez Aguilar et al., 2012; Oliveira et al., 2012; Pollard and Gómez, 2012). Handball alongside basketball usually show a higher ratio of home wins than in other team sports (Pollard et al., 2017) with slight differences due to league level, gender, or country (Pollard and Gómez, 2012). According to Pic (2018a), home advantage in handball exists mainly due to better performance of the home team in critical moments of the game.

Schwartz and Barsky (1977) had suggested a systematic theoretical framework which consists of three, mostly physical and external oriented, factors as the cause of home advantage. These factors were learning (i.e., familiarity with the playing field), traveling, and the fans. Later, Courneya and Carron (1992) expended this framework adding internal and psychological factors. Pollard (2008) summarized the literature into eight different factors: crowd influence, travel fatigue, familiarity, referees bias, territoriality, special tactics, psychological factors, and regulations. While all the above motioned factors are considered to influence the home advantage effect to some degree, contradictive findings do exist. For example, in contrast to Pollard (1986) and Agnew and Carron (1994) who found crowd size and crowd density to impact home advantage, Wolfson et al. (2005) found no such impact existed during the course of the 2002/2003 English premiere league season. The latter further noted that the influence of the crowd on the competitors’ performance is also difficult to establish. In a study conducted in 2021 during the global Covid19 pandemic, Wunderlich et al. (2021) found that while the crowd influences some elements of the game such as referee decisions and home team opportunities, it has no significant effect on home advantage in general, probably due to the dominance of other factors. In this vein, Fischer and Haucap (2020) have postulated that the crowd impact on home advantage should be considered negligible compared to the importance of psychological factors. While each factor also has a psychological effect on the athletes’ performance, the term *psychological factors* refers to the athletes’ readiness for the game and their perception of what is defined as success in it. In the context of familiarity, Pollard (1986, 2002) and Clarke and Norman (1995) validated the importance of this factor while other findings on the subject were inconclusive (e.g., Loughead et al., 2003; Watson and Krantz, 2003; Levental, 2015).

As aforementioned, the existence of home advantage is well established in the literature. Nonetheless, only limited attention was given to the beliefs and perceptions of the key characters involved in this phenomenon (i.e., athletes, coaches, officials, and fans) and its compliance with the known models (Fothergill et al., 2014). Moreover, merely a few scholars have compared these beliefs and perceptions among those groups. The perception of the home advantage and its causes is essential as it affects its prevalence. Staufenbiel et al. (2015) found that game location, home or away, influence coaches to adopt different tactics and play styles and even hold different expectations of the desired outcome of the competition. Consistently, Staufenbiel et al. (2018) showed that the rate of home advantage in sports increases with age, indicating that the expectations of better outcomes at home games are a developed trait. That is, being aware of the existence of a home advantage contributes to the formation of the phenomenon.

The research on perceived factors of home advantage is centered on athletes and fans. In general, athletes reported fan support, travel factors, and familiarity with the court as the most important variables affecting home advantage (Bray and Widmeyer, 2000). Indeed, in rugby, McGuckin et al. (2015) found that players perceive fans, especially family relatives and friends, as well as short travel and field familiarity as important facilitators of home advantage. In addition, the rugby players suggested sleeping arrangements and familiarity with the weather as crucial variables. However, among these various factors, field familiarity was found to be perceived as the most important factor for athletes as well as coaches (Bray et al., 1998; Gayton et al., 2001; Fothergill et al., 2014).

On the other hand, fans perceive their own involvement as the main factor in home advantage (Smith, 2005). English soccer fans ranked (out of 5) the fans’ support as the most important factor (4.40), then, familiarity with the pitch (4.07), territory (3.89), travel aspects (3.76), and referees bias (2.72; Wolfson et al., 2005). The authors concluded that these perceptions represent the fans’ beliefs in motivating their team and undermining the opposing team performance. On the other hand, fans perceive officials’ biases as less salient as they believe officials are more influenced by the teams’ ranking than the location of the game.

Being among the key characters themselves, officials take an active role in the game. However, in contrast to athletes, coaches, and fans, they are expected to remain neutral. Indeed, Goldschmied and Hochuli (2014) found that while the fans believe they have the most significant impact on the officials, the officials themselves believe the fans’ impact on their decisions is neglectable. Furthermore, all the officials in the study reported that while other officials are slightly affected by the fans, they are immune to such an effect (i.e., self-bias). Similar perceptions were found among officials and fans regarding the fans’ involvement in home advantage through their ability to affect athletes’ performances.

Anderson et al. (2012) compared key characters and their perceptions regarding home advantage. The research focused on soccer players, fans, and referees in England. The authors hypothesized that (a) referees will provide more importance to spacial factors such as travel aspects and familiarity with the field, and the fans, (b) players will attribute home advantage to the effect fans have on the officials, and (c) fans will attribute success to themselves through supporting their team and intimidating the opponents. The results revealed that all the participants perceived the home environment category as the most salient compared to the officials’ and players’ condition categories. As expected, fans, more than the officials and the athletes, have reported the motivation they provide to their players as the most vital factor in home advantage. Maintaining their neutral position as hypothesized, referees reported their involvement in home advantage as marginal. Anderson et al. (2012) have summarized that, despite minor differences, overall, the groups presented relatively similar perceptions of the factors leading to home advantage.

Embracing Anderson et al.’s (2012) research, this study’s purpose was to compare home advantage perceptions of key characters. However, this study expanded Anderson et al.’s (2012) inquiry in several aspects. First, while Anderson et al. (2012) investigated soccer players, referees, and fans; this study has further investigated an additional main character in sport, the coach. Second, Anderson et al. (2012) focused on home advantage perceptions in soccer (high profile sport being played in an open field) when this study centered on handball (medium profile sport being played in a closed court). Third, this study focused on the highest level of expertise: elite players, coaches, referees, and fans of elite teams rather than on semi-professional athletes and county level referees.

Four hypotheses were formed based on the literature aforementioned. Initially, fans were hypothesized to perceive their own related factor, crowd, as significantly more important in comparison to the other study participants. In the same vein, players were hypothesized to perceive their related factors (i.e., familiarity, travel, territory, and psychological attributes) as significantly more important in comparison to the other participants. Third, compared to others, coaches were hypothesized to emphasize factors such as travel and referees that are external to their control. Last, the referees were hypothesized to rank their own contribution to the home advantage lower than the other participants.

## Materials and Methods

### Participates

A total of 232 people participated in this study. Half were handball fans (*N* = 117) and half were combined of professional players, coaches, and referees (*N* = 59, 26, and 30, respectively). Specifically, fans from six different teams were randomly sampled at the arena’s entrance prior to their team’s game. The Fans’ age ranged from 18 to 65 (*M* = 33.06, *SD* = 13.83) and they were both males and females in gender (91 and 26, respectively). Players from four teams participated (Mage = 26.00, *SD* = 7.86). These players had, on average, more than 7 years of experience (*M* = 7.47, *SD* = 5.51) in the Israeli premier league; 20 of them have won titles (i.e., a national championship and/or cup) and 25 played in the Israeli national team (an average of 22 international performances). The coaches, on average, were 43.23 years old (*SD* = 7.05), had 8.73 year of experience in the Israeli premier league (*SD* = 7.17) and had 11.20 years of experience as players (*SD* = 7.63). In addition, 12 of them played for the Israeli national team (an average of 50.85 international performances). The referees were 39.5 years old on average (*SD* = 13.68). All referees were from the premier league with mean experience of 9.87 years (*SD* = 9.10). Six referees had a massive international experience (*M* = 47.83, *SD* = 23.19 international performances).

### Instrumentation

Based on Pollard’s (2008) conceptualization, seven factors were used to underlie the home advantage phenomenon: familiarity, crowd, travel, referees’ bias, territoriality, psychological, and tactical. Hence, participants were asked to grade each factor’s contribution to home advantage on a Likert scale from 1 (not at all) to 7 (very much). Examples are “to what degree is familiarity with the court important to home advantage (e.g., being familiar with the court size, the bounciness of the parquet)” and “to what degree is the crowd important to home advantage (e.g., encouragement and reinforcement of the home team, commitment facilitation).” Such single-item scales (i.e., single item for each dimension) are based on high face and ecological validity and have been found to be appropriate as dependent variables measures (see extensive elaboration on this issue in Tenenbaum et al., 2007). Since the study was conducted in Israel the items were provided in the Hebrew language.

### Procedure

Following the IRB approval protocol, all participants signed their participation consent prior to anonymously (a) providing their demographic data and (b) replying to the home advantage items. Fans were approached prior to official league games. Players were approached through their clubs and completed their participation prior to a team practice. Some coaches were addressed personally while others were addressed while participating in a professional coaching seminar. Referees completed their participation during a professional refereeing seminar.

### Statistical Analysis

Descriptive statistics were used to capture the importance of each of the home advantage factors per group. Correlational analysis was used to examine the relationship between age and the perceived contribution of each of the home advantage factors. Four group-rest (i.e., compared to the rest of the groups) MANOVAs (i.e., players-rest; coaches-rest, fans-rest, and referees-rest) followed by factors ANOVAs were conducted to uniquely identify the differences between each target-group and all the other handball involved personnel as a whole. Last, a MANOVA followed by factors ANOVAs with Bonferroni *post hoc* analysis was conducted to capture the differences between the four target groups on each of the factors. Effect Size (ES) calculations, based on Cohen’s d formula, were used to further demonstrate those differences.

## Results

Descriptive statistics revealed that the crowd was perceived as the most important factor in the home advantage phenomenon (*M* = 5.7). Both traveling and referees’ bias were perceived as the least influential factors to home advantage (*M* = 3.4). No statistical differences (*p* = 0.19) were found between genders for any of the home advantage factors. A significant positive correlation (*r* = 0.25) and a significant negative correlation (*r* = −0.23) were found between the tactics and territory factors, respectively, and the fans’ age. The total and categorical results for each of the home advantage factors are presented in Table 1.

**TABLE 1:** Total and categorical descriptive statistics for the home advantage factors.

Factor/Group	Crowd M (SD)	Famil M (SD)	Psych M (SD)	Terr M (SD)	Tactics M (SD)	Ref M (SD)	Travel M (SD)
T	5.7 (1.4)	5.2 (1.3)	5.0 (1.5)	4.8 (1.6)	3.7 (1.8)	3.4 (1.5)	3.4 (1.6)
F	6.1 (1.1)	5.5 (1.2)	5.4 (1.2)	4.8 (1.4)	4.4 (1.7)	3.4 (1.5)	2.9 (1.4)
P	5.7 (1.3)	5.1 (1.2)	4.6 (1.6)	5.5 (1.4)	2.7 (1.5)	3.7 (1.3)	3.9 (1.5)
C	5.1 (1.4)	5.2 (0.9)	5.1 (1.5)	4.8 (1.5)	3.5 (1.6)	4.1 (1.6)	4.1 (1.7)
R	4.7 (1.8)	4.2 (1.9)	4.4 (1.8)	3.8 (1.8)	2.9 (1.6)	2.3 (1.3)	3.9 (1.7)

*T, total; F, fans; P, players; C, coaches; R, referees.*

The first hypothesis centered on the fans’ perception in comparison to the rest in general and each personnel group separately. The MANOVA results revealed significant differences between the fans and the others for the crowd factor [*F*(1,230) = 20.02, *p* < 0.001; ES = 0.59]. Furthermore, the *post hoc* analysis revealed that the fans ranked this factor significantly higher than the coaches and referees (*p* < 0.01; ESs = 0.86 and 1.08, respectively) but not than the players. The fans significantly ranked higher than the others the psychological factor, the tactical factor (*p* < 0.001 for both; ESs = 0.55 and 0.91, respectively) and the familiarity with the court factor (*p* < 0.01; ES = 0.46). In contrast, the fans significantly ranked lower the travel factor (*p* < 0.001; ES = 0.74). No difference was found between the fans and the others on both the territory and the referees’ bias factors. The results of the MANOVA, ANOVAs and *post hoc* analyses are presented in Table 2.

**TABLE 2:** The MANOVA, ANOVAs, and *post hoc* analyses results of the *fans* category in comparison to the *players*, *coaches*, and *referees* categories.

Factor/Group	Crowd	Famil	Psych	Terr	Tactics	Ref	Travel
MANOVA	Wilk’s Lambda: *F*(7,224) = 19.61, *p* > 0.001, η2 = 0.38						

Fans-rest	*F* = 20.2	*F* = 12.2	*F* = 17.40	*F* = 0.38	*F* = 48.17	*F* = 0.19	*F* = 31.03
ANOVAs	*P* = 0.001	*P* = 0.000	*P* = 0.000	*P* = 0.54	*P* = 0.000	*P* = 0.67	*P* = 0.000

Fans-Players *Post hoc*	*P* = 0.44	*P* = 0.47	*P* = 0.002	*P* = 0.014	*P* = 0.000	*P* = 0.76	*P* = 0.000

Fans-Coaches *Post hoc*	*P* = 0.002	*P* = 1	*P* = 1	*P* = 1	*P* = 0.038	*P* = 0.098	*P* = 0.001

Fans-Referees *Post hoc*	*P* = 0.000	*P* = 0.000	*P* = 0.002	*P* = 0.006	*P* = 0.000	*P* = 0.004	*P* = 0.004

*T, total; F, fans; P, players; C, coaches; R, referees.*

Secondly, it was hypothesized that, in comparison to other participants, the players will perceive their related factors (i.e., familiarity, travel, territory, and psychological attributes) as significantly more important. Significant differences were obtained from the MANOVA analysis for the territory (*p* < 0.001; ES = 0.59) and travel (*p* < 0.01; ES = 0.44) home advantage factors. However, players, coaches, and referees did not differ in familiarity and psychological factors’ ranking (while, as aforementioned, the fans significantly graded these factors as high in their importance).

Coaches were hypothesized to emphasize factors that are external to their control such as the travel and the referees. The statistical analysis yielded several interesting findings. First, coaches ranked the referees’ bias higher than all the other participants (*p* = 0.01; ES = 0.54). Second, coaches ranked the importance of the travel in home advantage significantly higher (*p* = 0.001; ES = 0.84) than the fans. In contrast, coaches ranked the contribution of the crowd to home advantage significantly lower than the fans (*p* = 0.002; ES = 0.86). No difference was obtained between the coaches and the rest in familiarity, territory, psychosocial and tactical factors.

Last, it was hypothesized that the referees will rank their own contribution to the home advantage lower than the other participants. The results revealed that referees significantly under ranked (*p* < 0.001; ES = 0.84) their contribution to the phenomenon. Furthermore, referees also under ranked all the other home advantage factors: travel (*p* = 0.05; ES = 0.38), familiarity (*p* < 0.001; ES = 0.93), psychological attributes (*p* < 0.01; ES = 0.53), territory (*p* < 0.001; ES = 0.82), and tactics (*p* < 0.01; ES = 0.53).

## Discussion

**FIGURE 1:**
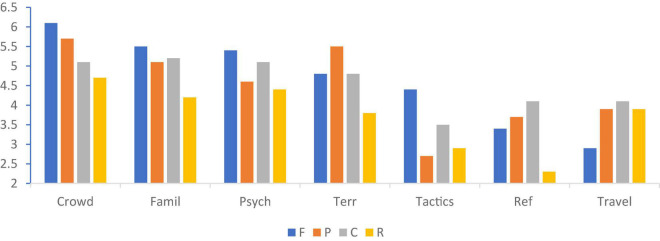
Home advantage factors ratings by group. F, fans; P, players; C, coaches; R, referees.

The purpose of the study was to compare the perceptions of key characters in sports (i.e., fans, players, referees, and coaches) on the roles various factors play in the home advantage phenomenon. The main findings from the study indicated that (a) across groups and in general, the crowd was considered to play the most important role in home advantage with familiarity, psychology and territory also playing important roles, (b) there are differences among fans, players, referees, and coaches in their perceptions of what contributes to home advantage (see Figure 1), and (c) that these differences are mainly attributed to the self-serving bias (Shepperd et al., 2008). Meaning that the groups want to protect their ego and either feel proud or avoid feeling embarrassed (Leary, 2007).

Previous studies in the area confirmed that the home advantage phenomenon exists across sports, levels, and countries (Jamieson, 2010). Moreover, as Pic (2018b) suggests, home advantage should also be considered as part of planning training tasks for maximizing performance. However, it is less clear what factors contribute the most to the home advantage phenomenon. Some important factors that were examined included crowd, familiarity, psychology, territory, tactics, referees’ bias, and travel distance (Pollard, 2008). Regardless of which is the most important factor, it seems that all play a role in contributing to the home advantage. One way to learn more about the phenomenon and expand the knowledge in the area, is to examine the perceptions of key characters involved in sports about the role various factors play in the home advantage (Anderson et al., 2012).

The findings from the current study revealed that crowd, familiarity, psychology, and territory are all perceived to be important in the home advantage (i.e., average scores higher than 4.00 across all groups). While tactics, referees and travel seem to be perceived to play a relatively minor role in the home advantage, with some minor differences among groups. This partially supports previous research in which fan and familiarity were also found to be among the most important factors in home advantage as perceived by players (Bray and Widmeyer, 2000; McGuckin et al., 2015). However, travel which was perceived to be one of the least important factors in this study, was perceived to be important in previous studies with players (Bray and Widmeyer, 2000; McGuckin et al., 2015). This may be due to the relatively small size of the state of Isreal (i.e., no flights or hotels are needed). A major difference in the current study is that, in addition to players, other key characters participated in the study (i.e., fans, coaches, and referees). Indeed, in one of the only other studies that compared several key characters (i.e., players, fans, and referees), crowd was the most important factor across groups, similar to the current study (Anderson et al., 2012).

This is an interesting and important finding, especially when compared to studies that examined actual effects and not perceived effects as in the current study. Specifically, the actual importance (not perceive importance) of the crowd in home advantage studies seems to be relatively minor compared to other factors such as psychological, familiarity, and territorial factors (Fischer and Haucap, 2020; Wunderlich et al., 2021). Thus, future studies should examine what contributes to the differences between actual and perceived contribution to the home advantage phenomenon.

Another main finding was the differences between groups in their perceptions of the contributing factors. In general, it was hypothesized that fans will perceive their own related factor, crowd, as significantly more important in comparison to the other study participants all together. This hypothesis was statistically supported. Similar to the other groups, fans ranked crowd as the most important factor. However, when separately comparing the average ratings of the crowd factor, fans ranking was significantly higher than the coaches and referees, and descriptively higher than players. This confirms the self-serving bias hypothesis, indicating that fans feel that they are the most important factor in the home advantage (Wolfson et al., 2005). This also supports previous research that examined fans perceptions on the contributing factors to the phenomenon compared to other key characters (Anderson et al., 2012). Furthermore, this finding aligns with theories of self-motives and emotions, including self-enhancement and self-verification (Leary, 2007). Specifically, a sub aspect of self-enhancement is self-serving attribution, and is explained as people’s tendency to attribute positive outcomes to themselves to feel proud and motivated (Leary, 2007) and avoid feeling shame or embarrassment, which is also a motivational factor (Graham, 2020).

With regards to the other key characters, findings revealed that players only rated territory higher and coaches only rated referees’ bias higher than the other groups (both provide a partial support to our hypotheses). Regarding referees, they (a) rated most of the factors lower than the other characters; (b) rated the referees’ bias factor as the least important factor contributing to home advantage; and (c) rated, as hypothesized, the referees’ bias factor significantly lower compared to the other participants. Together, these findings support the aforementioned self-serving bias (Leary, 2007; Anderson et al., 2012). The groups wanted to increase their positive feelings by rating attributions that they can control relatively low (e.g., coaches and players rating tactics the least important), compared to factors that they have less control (e.g., coaches rating familiarity as the most important).

It is important to note that a possible limitation of the study is that the factors were provided to the participants, and they could not add additional factors. Future studies should explore other possible factors and allow participants to generate the factors so that they won’t be influenced by the factors provided. In addition, except for the fans group, the other groups were relatively small and were from only a few teams, and in general, this study was specifically about handball. Thus, future studies need to examine and compare the perceptions in additional sports and with more participants from each group, especially, because for example the number of fans that attend handball games is relatively small in Israel compared to basketball and soccer.

## Conclusion and Implications

The current study is one of the first studies that examined and compared the perceptions on home advantage of four key characters (i.e., fans, players, coaches, and referees) in sports (Anderson et al., 2012). Findings indicated that there are differences among characters in their perceptions of the most important contributing factors to home advantage. More importantly, it seems that self-serving attribution is the reason for these differences. To confirm this, future studies should explore not only perceptions but also the rationale or reason for the perceptions. This will shed light on the mechanisms responsible for the differences among characters. Furthermore, studies should also explore the effects of the perceptions on the characters’ behaviors and attitudes (Staufenbiel et al., 2015). Generating more knowledge in the domain can have practical implications for the key characters and other characters (e.g., media) by developing ways to prevent or cope with factors contributing to the home advantage when playing away and finding ways to add to the factors that contribute to the home advantage when playing at home.

## Data Availability Statement

The raw data supporting the conclusions of this article will be made available by the authors, without undue reservation.

## Ethics Statement

The studies involving human participants were reviewed and approved by Tel-Hai Academic college. The patients/participants provided their written informed consent to participate in this study.

## Author Contributions

LG collected and analyzed the data and contributed to the write-up. OL and IB contributed substantially to the write-up of the manuscript. All authors contributed equally to the article and approved the submitted version.

## Conflict of Interest

The authors declare that the research was conducted in the absence of any commercial or financial relationships that could be construed as a potential conflict of interest.

## Publisher’s Note

All claims expressed in this article are solely those of the authors and do not necessarily represent those of their affiliated organizations, or those of the publisher, the editors and the reviewers. Any product that may be evaluated in this article, or claim that may be made by its manufacturer, is not guaranteed or endorsed by the publisher.
